# Parachute mitral valve associated with reticular chordae tendineae in an adult: case report

**DOI:** 10.1186/s13019-021-01448-4

**Published:** 2021-04-09

**Authors:** Qun-Jun Duan, Cui-Ting Duan, Ai-Qiang Dong, Hai-Feng Cheng

**Affiliations:** 1 Department of Cardiovascular Surgery, Second Affiliated Hospital of Zhejiang University, School of Medicine, #88 Jiefang Road, Hangzhou, 310009 China; 2 Department of Nursing, Hangzhou Medical College, Hangzhou, China

**Keywords:** Case report, Parachute mitral valve, Chordae tendineae, Valve replacement, Mitral valve anomaly

## Abstract

**Background:**

Parachute mitral valve with reticular chordae tendineae is an extremely rare anomaly.

**Case presentation:**

We present a case of parachute mitral valve associated with distinctive reticular chordae tendineae in an adult. It was diagnosed from the echocardiogram. The patient was referred for surgery. Valve analysis showed thickened mitral valve leaflets and commissures. The chordae tendinae were lengthy and thick. All the chordae tendinae merged into a solitary papillary muscle. A distinctive reticular fibrous tissue was found on mitral valve apparatus as the chordae tendinae intermixed each other. The only functional communication between the left atrium and the left ventricle was through the reticular spaces. This anomaly was considered to be unrepairable and was replaced with a mechanical valve.

**Conclusions:**

An extremely rare and unique case of parachute mitral valve associated with reticular chordae tendineae was reported. Mitral valve replacement is a reasonable choice in patients with parachute mitral valve with reticular chordae tendineae.

**Supplementary Information:**

The online version contains supplementary material available at 10.1186/s13019-021-01448-4.

## Background

Parachute mitral valve (PMV) is characterized by a unifocal attachment of the mitral chordae tendinae to a single papillary muscle [1, 2]. Very few adult patients with this isolated anomaly are reported [3–7]. The rarity and complexity of parachute valves, as well as their occurrence in infants and children, has stimulated great interest and fascination in its surgical management. We report a case of PMV associated with distinctive reticular chordae tendineae in an adult. To the best of our knowledge, this is the first case of PMV associated with reticular chordae tendineae in an adult reported in literature.

## Case presentation

A 36-year old man developed exertional dyspnea and fatigue of 2 months duration. There was no history of orthopnea or paroxysmal nocturnal dyspnea. Physical examination revealed a well-built man with supine right upper arm blood pressure of 135/76 mmHg, regular pulse rate of 88 beats/min, and no evidence of heart failure. Precordial examination showed normal heart sounds, a 3/6 diastolic rumble murmur. No opening snap or third sound was audible. An electrocardiogram revealed sinus rhythm with a heart rate of 86beats/min, left atrial overload and normal atrio-ventricular conduction. The chest skiagram showed mild left atrial prominence. Routine Laboratory tests showed no anaemia, liver dysfunction or renal dysfunction. He underwent detailed transthoracic echocardiographic examination.

**Fig. 1:**
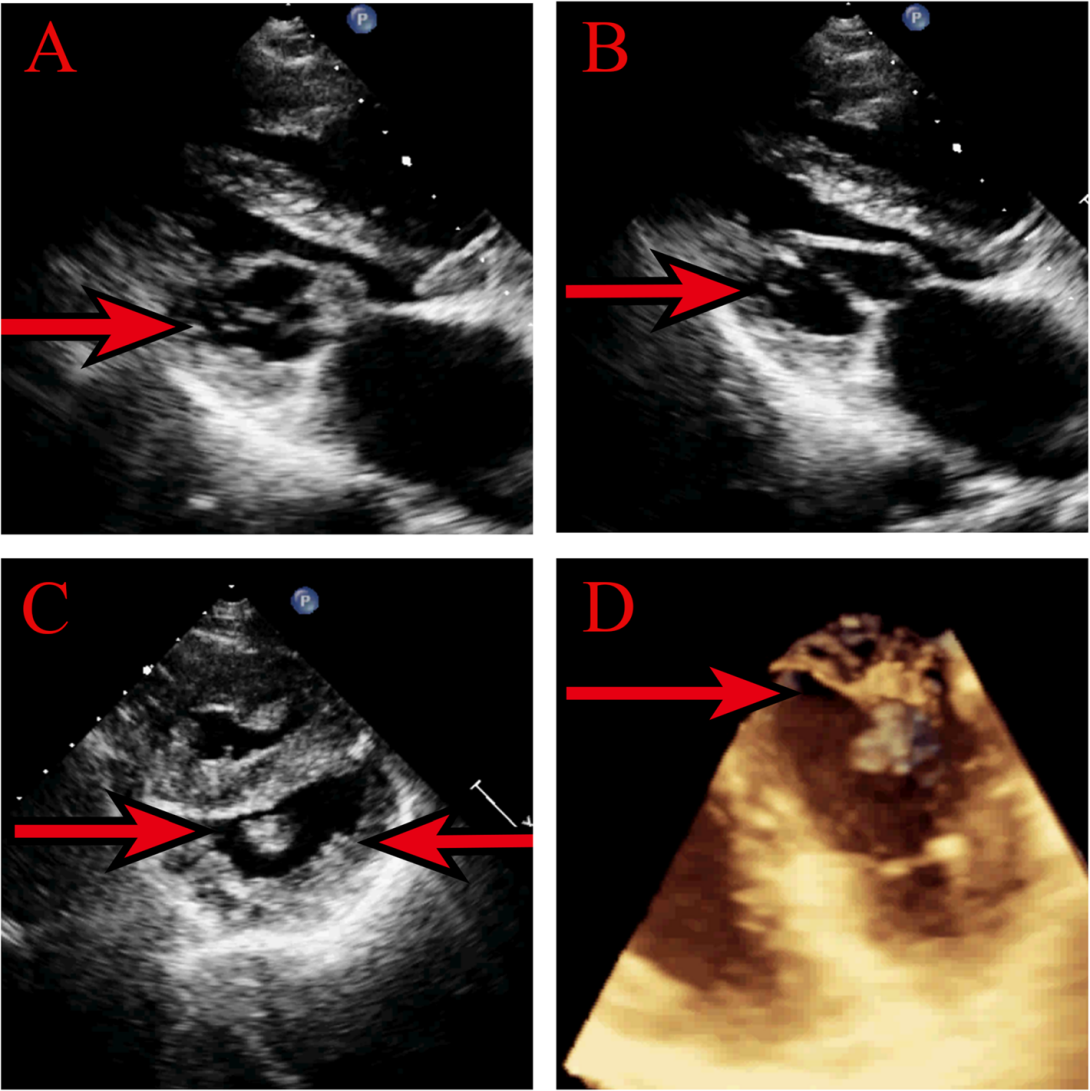
**a**. Transthoracic two-dimensional echocardiography showing abnormal chordae tendineae with reticular structures (arrow) and slightly thickened mitral leaflets in systole. **b**. Parasternal long-axis view showing pear-shaped mitral orifice during diastole (arrow) with chords attached to the posteromedial papillary muscle, with the leaflets forming the larger base of the pear and the chordae tendinae the apex. **c**. A single large papillary muscle in short-axis format was detected. A small muscular ridge or trabecula was present at the location of anterolateral papillary muscle without any chordal attachment. **d**. Reticular chordae tendineae with scattered holes among them was confirmed in 3D transesophageal echocardiography

Echocardiogram revealed dilated left atrium, normal left ventricle and normal left ventricular function. Mitral valve area by planimetry and the pressure half-time method was 1.02 cm^2^ with a trans-mitral peak and mean gradient of 9 and 5 mmHg respectively. Abnormal chordae tendineae with reticular structures attached to a solitary papillary muscle originating from the posteromedial wall was detected (Fig. 1 a, b, c). A small muscular ridge or trabecula was present at the location of anterolateral papillary muscle without any chordal attachment (Fig. 1c). An additional movie file shows this in more detail [see Additional file 1]. No other congenital heart anomalies were identified. A 3D transesophageal echocardiography was performed. Reticular chordae tendineae with scattered holes among them was confirmed (Fig. 1d). An additional movie file shows this in more detail [see Additional file 2]. Thus, the final diagnosis was isolated parachute mitral valve with reticular chordae tendineae and severe mitral stenosis, without any other congenital heart anomalies.

**Fig. 2:**
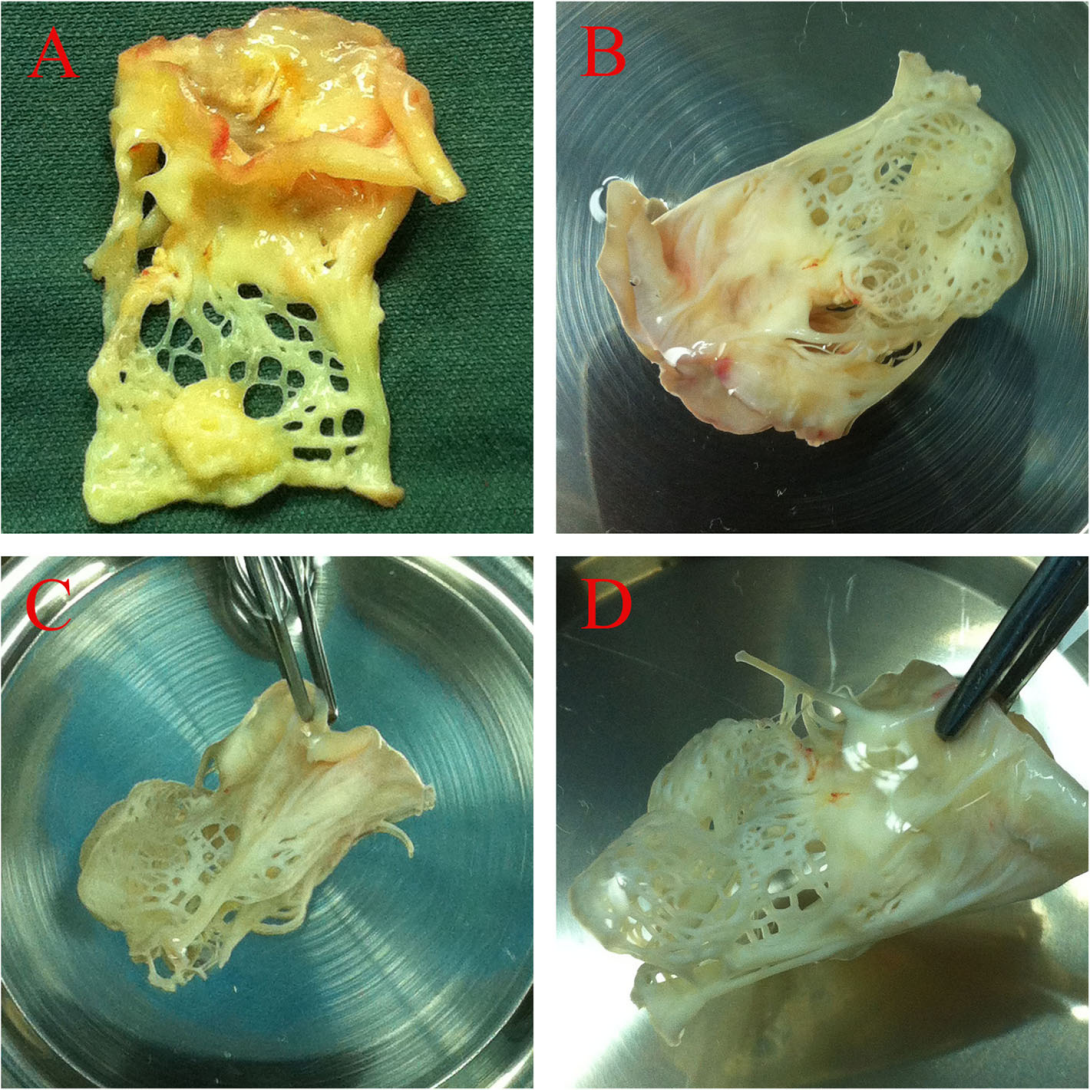
**a**. The chordae tendinae were lengthy and thick. The leaflets were slightly thichened. A distinctive reticular fibrous diaphragm with scattered holes was found on mitral valve apparatus as the chordae tendinae intermixed each other. B, C, D. The valve was displayed in saline and was shown in different views. The only functional communication between the left atrium and the left ventricular was through the reticular spaces. Noting that the arrow marked small chodae tendinae in Fig. 2**a, c, d** did not attach to any other small papillary muscle or the ventricle wall. It was actually attached to the reticular fibrous diaphragm. The attachment point was marked with asterisk mark in Fig. 2**a**. This small chordate tendinae was inadvertently cut from the fibrous diaphragm during the resection of mitral valve

The patient was referred for surgery. The operation was performed through a minimally invasive right thoracotomy with the use of cardiopulmonary bypass. Through an interatrial approach, the mitral annulus, leaflets, chordae tendinae, and papillary muscles were exposed. Valve analysis showed two thickened mitral valve leaflets and commissures, but all the chordae tendinae merged into a solitary papillary muscle. It presented as a funnel-type structure. The chordae tendinae were lengthy and thick. A distinctive reticular fibrous diaphragm with scattered holes obstructing the valvular orifice was found on mitral valve apparatus as the chordae tendinae intermixed each other (Fig. 2). An additional movie file shows this in more detail [see Additional file 3]. The valve naturally was deformed. These anomalies, coupled with their convergent papillary insertion, resulted in restricted leaflet mobility, thus creating a stenotic mitral valve as the leaflets were closely apposed, greatly reducing the effective mitral orifice area. It was seen that the only functional communication between the left atrium and the left ventricular was through the reticular spaces. These spaces did not allow free outflow of blood from the left atrium. The patient was implanted with a #27 St Jude’s prosthesis in mitral position.

The postoperative recovery was uneventful. The patient was discharged 7 days after surgery. At the 5-year follow-up examination, the patient was in good health.

## Discussion

Parachute mitral valve is a very rare congenital mitral valve anomaly which may remain asymptomatic in life-time or may present with mitral valve stenosis or regurgitation or both. It is characterized by unifocal attachment of the chordae tendineae of both leaflets to a single papillary muscle. It is highly associated with other congenital heart anomalies. Isolated PMV in an adult is very rare, while the presence of a reticular chordae tendineae--as in the present case--is extremely exceptional.

In the developmental process of mitral valve, PMV occurs due to disturbed delamination of the anterior and posterior parts of the trabecular ridge [4]. These parts normally form anterolateral and posteromedial papillary muscles, respectively, between the 5th and 19th week of gestation, thereby forcing these embryonic predecessors of the papillary muscles to condense into a single papillary muscle [8, 9]. The chordae tendinae in PMV are often underdeveloped and hence short, thick, and adherent, causing decreased mobility of the valve leaflets and reducing the size of mitral orifice [9]. Narrowing of the interchordal spaces due to unifocal insertion results in a smaller mitral orifice. The interchordal spaces in the present case are in a unique reticular shape and result in severe stenosis of mitral valve.

Although adult patients with isolated PMV usually present with dyspnea, PMV may be incidentally diagnosed during echocardiography. Such patients generally require no medical or surgical treatment. Mitral valve surgery when feasible needs to be performed only in those patients with hemodynamically significant stenosis or regurgitation [9]. It is well known that mitral valve repair is superior to mitral valve replacement in terms of long-term prognosis. However, the results of the mitral valvuloplasty for PMV in the adult are not completely clarified [10, 11]. Repair is feasible in case there is predominant regurgitation with well-developed chords. Replacement is a reasonable choice when the leaflets are thick and severely stenotic.

## Conclusions

We reported an extremely rare and unique case of parachute mitral valve associated with reticular chordae tendineae in an adult. Mitral valve replacement is a reasonable choice in patients with this anomaly when the leaflets are thickened and severely stenotic.

## Supplementary Information

**Additional file 1:.** Abnormal chordae tendineae which was attached to a solitary papillary muscle originating from the posteromedial wall was detected. A small muscular ridge or trabecula was present at the location of anterolateral papillary muscle without any chordal attachment.

**Additional file 2:.** 3D transesophageal echocardiography showed reticular chordae tendineae with scattered holes among them.

**Additional file 3:.** Valve analysis showed all the chordae tendinae merged into only 1 major papillary muscle. The chordae tendinae were lengthy and thick. A distinctive reticular fibrous diaphragm with scattered holes obstructing the valvular orifice was found on mitral valve apparatus as the chordae tendinae intermixed each other. The only functional communication between the left atrium and the left ventricular was through the reticular spaces.

## Data Availability

The datasets used and/or analysed during the current study are available from the corresponding author on reasonable request.

## Abbreviations

PMV: Parachute mitral valve
